# A novel vector for magnetic resonance imaging-guided chemo-photothermal therapy for cancer

**DOI:** 10.3389/fonc.2022.972082

**Published:** 2022-10-17

**Authors:** Ji chuan Kong, Yang Li, Wang Ma, Yu rong Du, Lei Liu, Tian tian Qu, Shuo shuo Liu, Meng li Wang, Wei Dou

**Affiliations:** ^1^ Medical College, Henan Polytechnic University, Jiaozuo, China; ^2^ Department of Oncology, The First Affiliated Hospital of Zhengzhou University, Zhengzhou, China

**Keywords:** humic acids, nanocarrier, magnetic resonance imaging, combination therapy, HA-Gd

## Abstract

As an effective strategy for oncotherapy, developing efficacious drug delivery systems for cancer combination therapy remains a major challenge. To improve nanodrug biocompatibility and composite function facilitating their clinical conversion application, a novel nanocarrier was presented by a facile method through conjugating humic acid with gadolinium ions to synthesize HA-Gd with good biocompatibility and dispersity. HA-Gd exhibited high photothermal conversion efficiency up to 38%, excellent photothermal stability, and high doxorubicin (DOX) loading capacity (93%) with pH-responsive release properties. HA-Gd loading DOX showed a combined chemo-photothermal inhibitory effect on tumor cells. Compared with lipid-DOX, HA-Gd-DOX had a more significant inhibitory effect on tumor growth and fewer side effects. T_1_-weighted MRI contrast toward tumor tissue provided HA-Gd with an MRI-based cancer diagnosis. This study revealed the great potential of humic acids as a novel vector for developing more drug carriers with desirable functions for clinical anticancer therapy.

## Introduction

Cancer is the second leading cause of death globally, with an increasing incidence due to the world’s aging population (1). Chemotherapy is the most commonly used method for cancer treatments. However, many chemotherapeutic molecules possess a generalized mechanism of action that allows them to act on healthy and cancer cells (2). Doxorubicin (DOX) has obvious cardiotoxicity, myelosuppression, hepatotoxicity, nephrotoxicity, and other adverse reactions in the process of chemotherapy, with cardiotoxicity being the most serious and most common, which results in poor prognosis in clinical application (2–5).

In the last decade, nano-drug delivery systems have been considered as a promising platform for cancer therapy because nano delivery vehicles have unique abilities such as enhanced permeation and retention effect (EPR) to overcome biological barriers, high loading efficiency, improved drug accumulation, targeted lesion sites, controlled drug release, and prolonged pharmacological effects (6–8). Recently, a variety of nano materials such as gold nanostructures (nanocages, nanoshells, and nanorods) (9–11), polymers (12–14), carbon nanomaterials (carbon nanotubes, graphene, nanodiamond, and fullerene) (15–18), and others (19) were investigated as nanocarriers in an effort to enhance the therapeutic efficiency of tumor. However, such inorganic nanomaterials have low solubility, poor dispersion in aqueous solution, poor biocompatibility, and easy agglomeration, which hindered their clinical transformation application. Although organic nanomaterials such as PEG, PEI, liposome, and chitosan have good biocompatibility and solubility in aqueous solution, the lack of imaging and therapeutic capabilities limits their clinical applications. Most researchers coated inorganic nanometer materials with organic materials to form composite materials to improve the performance of materials, while the complex preparation technology was not conducive to mass production. Thus, it is necessary to develop new multifunctional vectors for better diagnosis and therapy of tumors.

Humic acid (HA), having organic large molecules, has emerged as a promising material due to its simple preparation, high biocompatibility, extreme dispersion properties, and colloidal stability (20, 21). The abundant functional groups on the surface of HA, such as carboxyl group, phenolic hydroxyl, alcoholic hydroxyl, methoxy, carbonyl, and quinone, result in HA molecules performing physiological activities (22), which are widely used in medicine and healthcare (23, 24). At the same time, HA also acts as a chelating agent to chelate metal ions to form complexes. The above-mentioned properties of HA make it promising for the delivery of drugs and metallic contrast agents. HA has been used as a photothermal agent due to its good photothermal conversion properties (25). However, few reports on using HA as a drug delivery system for tumor therapy have been found.

**Scheme 1:**
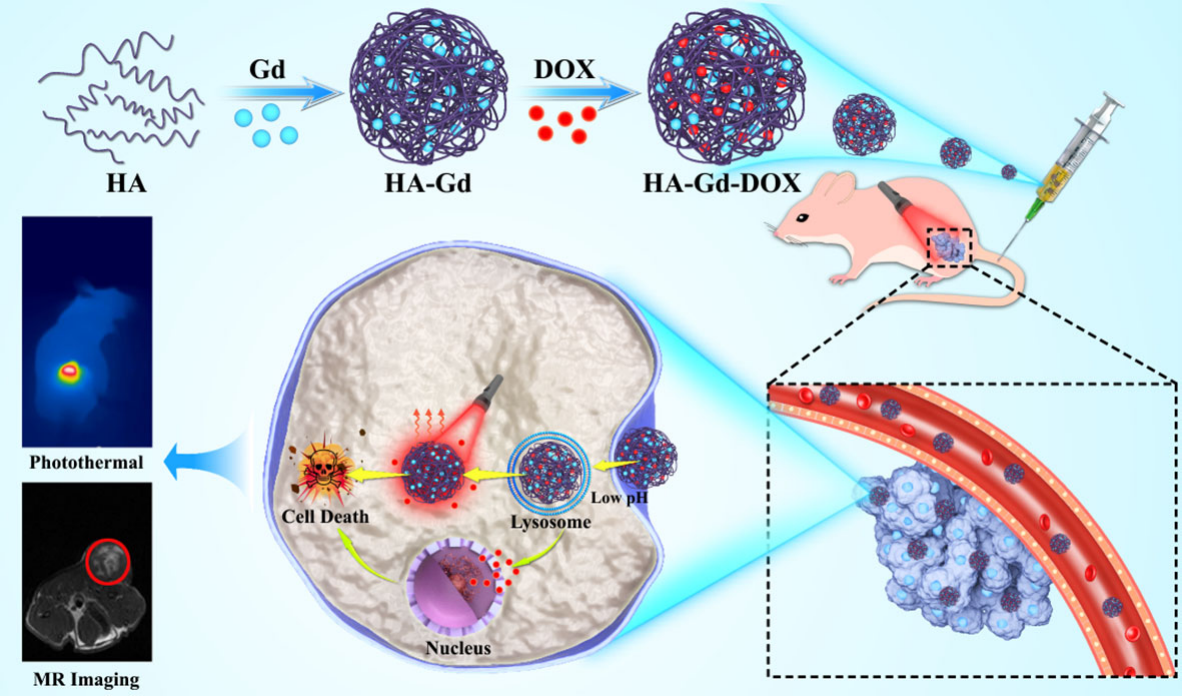
Schematic of the preparation of HA-Gd-DOX to implement photothermal/chemotherapy for cancer under the magnetic resonance imaging guide.

In this study, a novel diagnostic and therapeutic integrated nanodrug delivery system based on HA, Gd^3+^, and DOX was rationally designed for magnetic resonance imaging (MRI)-guided chemotherapy–photothermal combined therapy for cancer. HA and Gd^3+^ were complexed to prepare HA-Gd nanocarriers with porous nanostructures, and DOX was loaded to form the HA-Gd-DOX nano-drug delivery system. Upon near-infrared (NIR) laser irradiation, HA-Gd-DOX could exhibit high photothermal conversion efficiency and photothermal stability, heralding its important role as a photothermal therapy (PTT) agent. The pH value and photothermal effect could lead to the gradual release of DOX in tumors, resulting in good chemo-photothermal therapy with negligible side effects on normal tissues, and MRI of HA-Gd-DOX could be used for tumor monitoring. By simple preparation, novel nanomedicine could enable HA to have imaging-guided chemo-photothermal tumor therapy, and the physiological activity of HA could alleviate the cardiac damage caused by DOX and Gd; thus, HA-Gd-DOX could have a potential clinical application for tumor therapy (**Scheme 1**).

## Materials and methods

### Materials

All chemicals and reagents were used without further purification unless otherwise noted. Sodium humate (SH) powder was procured from Makelin Co. Disodium hydrogen phosphate (Na_2_HPO_4_), sodium dihydrogen phosphate ammonium (NaH_2_PO_4_), 1-ethyl-3-(3-dimethylaminopropyl) carbodiimide hydrochloride (EDC), N-hydroxysuccinimide (NHS), DOX, and nitric acid gadolinium [Gd(NO_3_)_3_] were purchased from Sigma-Aldrich and used as received. CCK-8 kits were purchased from Shanghai Aladdin Co. (Shanghai, China). Dulbecco’s minimum essential medium (DMEM) cell culture medium, penicillin, streptomycin, and fetal bovine serum (FBS) were bought from Gibco Invitrogen. Human lung adenocarcinoma A549 cells were purchased from the Chinese Academy of Sciences Cell Bank and cultured in DMEM supplemented with 10% fetal bovine serum and 1% penicillin/streptomycin in a humidified atmosphere at 37°C in 5% CO_2_. 4′,6-Diamidino-2-phenylindole (DAPI) was purchased from Aladdin Reagent Co., Ltd. (Shanghai, China). Calcein-AM and propidium iodide (PI) were obtained from Summus. Lipo-DOX was provided by the First Affiliated Hospital of Zhengzhou University. Water and all buffers were of Millipore grade and pretreated with Chelex 100 resin to ensure that the aqueous solution was free of heavy metal. BALB/c nude mice were purchased from Beijing Sibafu Biotechnology Co. Ltd. Animals were fed sterile water and unrestricted food. They were placed in a mouse room with standard conditions. All the experimental steps adopted in this experiment conformed to the experimental scheme approved by the Animal Experiment Center of Zhengzhou University.

### Characterizations

The morphology test of HA-Gd was carried out by a ZEISS GeminiSEM 300 scanning electron microscope (SEM). Energy-dispersive x-ray (EDX) elemental mapping was analyzed by Tecnai G2 F30 S-TWIN TEM. X-ray diffraction (XRD) patterns were obtained using a Rigaku Ultima IV powder diffractometer. The material composition was determined by x-ray photoelectron spectroscopy (XPS) analysis (E250, Thermo-Fisher Scientific, USA). UV−Vis spectra were measured by a UV−Vis spectrophotometer (UV-2600). The hydrodynamic diameter was measured by a Zeta-Sizer (NICOMP Nano-ZLS Z3000). Fourier transform infrared spectroscopy (FTIR) was acquired by the NEXUS670 FTIR spectrometer. Fluorescence images (FL) were acquired using a confocal laser scanning microscope (CLSM) (TCSSP5, Leica, Germany). Thermal images were obtained by monitoring the MG33 system with PTT. MRI was acquired on a 4.7-T small animal MR scanner (Bruker I-CON).

### Preparing and purifying HA

The HA sodium salt was dissolved in 0.01 M hydrochloric acid, and after sonication for 5 min, it was centrifuged at a high speed of 8,000 r/min for 15 min to remove insoluble matter. Acidic HA was then prepared by overnight dialysis and lyophilized for use.

### Preparing of HA-Gd

Two hundred milligrams of purified and acidified HA was dispersed into 300 ml of deionized water and sonicated for 5 min. Subsequently, 20 mg of Gd salt was dissolved in 5 ml of hydrochloric acid solution with pH 4.0, sonicated for 5 min to dissolve completely, and then added to HA solution. After stirring at room temperature for 2 h to fully react, the reaction mixture was transferred to a 14-kDa retention dialysis tube to remove free Gd^3+^, and lyophilized to obtain HA-Gd.

### Synthesis of HA-Gd-DOX

Two hundred milligrams of purified and acidified HA was dispersed into 300 ml of deionized water and sonicated for 5 min. Subsequently, 20 mg of Gd salt was dissolved in 5 ml of hydrochloric acid solution with pH 4.0, sonicated for 5 min to dissolve completely, and then added to HA solution, followed by 20 mg of DOX. After stirring for 2 h at room temperature to fully react, the reaction mixture was transferred to a 14-kDa retention dialysis tube to remove the free DOX and Gd^3+^, and lyophilized to obtain HA-Gd-DOX.

### Preparing HA-DOX

Two hundred milligrams of purified and acidified HA was dispersed into 300 ml of deionized water and sonicated for 5 min. Subsequently, 20 mg of DOX was added to the mixture. After stirring for 2 h at room temperature to fully react, the reaction mixture was transferred to a 14-kDa retention dialysis tube to remove the free DOX, and lyophilized to obtain HA-DOX.

### Calculation

The amount of unloaded DOX in the supernatant was measured by a UV-Vis spectrophotometer at 480 nm. The DOX loading content in the HA-Gd was calculated by [(M_1_ − M_2_)/M_3_] ×100%, where M_1_ represented the original weight of DOX added, M_2_ was the weight of DOX in the supernatant, and M_3_ was the weight of HA-Gd. The amount of loaded Gd^3+^ in HA-Gd-DOX was measured by inductively coupled plasma-mass spectrometry (ICP-MS).

### *In vitro* stimuli-responsive DOX release from the HA-Gd-DOX

PBS with different pH values (5.0 and 7.4) was selected as the release medium to study the pH-responsive release behaviors of HA-Gd-DOX. Briefly, 10 ml of PBS containing HA-Gd-DOX was added to a dialysis tube [molecular weight cutoff (MWCO) = 14 kDa] immersed in 100 ml of release medium under gentle stirring at room temperature. Release medium (2 ml) was collected at the given time intervals and 2 ml of fresh medium was supplied to the solution. The collected solution was used to measure the concentration of DOX *via* a UV-Vis spectrophotometer at 480 nm.

### Photothermal performance of HA-Gd

To quantitatively determine the thermal properties of HA-Gd nanocarriers after laser irradiation, HA-Gd aqueous solutions (1.0 ml) with different concentrations (0, 100, 150, 200, 250, 300, and 400 μg/ml) were placed in a quartz cuvette, and the photothermal conversion performance of HA-Gd was measured by irradiating an 808-nm laser with a power density of 1.0 W/cm^2^ for 5 min. HA-Gd solution was also irradiated with a laser (0.5, 0.8, 1.0, 1.2, 1.5, and 1.8 W/cm^2^) for 5 min. A thermocouple probe with an accuracy of 0.1°C was inserted into the HA-Gd aqueous solution, and the photothermal stability of HA-Gd was investigated by cyclic heating. The HA-Gd solution was irradiated with an 808-nm laser for 5 min per cycle, followed by cooling to room temperature. The photothermal conversion efficiency (*η*) was calculated by the formula in the appendix.

### Cell culture

Human lung adenocarcinoma A549 cells were cultured in normal DMEM culture medium with 10% FBS and 1% penicillin/streptomycin in 5% CO_2_ and 95% air at 37°C in a humidified incubator. Intracellular drug release research and the cellular uptake of HA-Gd-DOX nanodrugs were investigated using a confocal laser scanning microscope (CLSM). The tumor cells were seeded at a density of 1×10^5^ cells/well onto a 35-mm Petri dish and incubated at 37°C for 12 h. Then, HA-Gd, DOX, and HA-Gd-DOX solutions were diluted to a final concentration of 300 μg/ml (HA-Gd), and cells were incubated for 3 and 24 h at 37°C, with DMEM as the control group. The cell monolayers were washed three times with phosphate-buffered saline (PBS, pH 7.4) and fixed with 4% paraformaldehyde for 30 min. Nucleus staining was performed with DAPI. After washing with PBS, cells were observed by a confocal laser scanning microscope.

### *In vitro* cytotoxicity assays

The cytotoxicity was studied by CCK-8 assay using A549 cells. The cells were seeded into a 96-well cell culture plate at 5 × 10^3^ per well under 100% humidity, and were cultured at 37°C and 5% CO_2_. After 24 h of adherent incubation, cells were co-incubated with different concentrations of HA, HA-Gd, DOX, HA-Gd-DOX, and HA-DOX for 24 and 48 h. Then, each well was added into CCK-8 solution (10 μl, 2 mg/ml). After 2 h, a microplate reader was used to test the OD value at 450 nm. For PTT, some A549 cells were incubated with HA-Gd or HA-Gd-DOX for 1.5 h and then irradiated with 1.0 W/cm^2^ NIR light for 5 min. After further incubation, the cell viability was measured by CCK-8 assays.

### *In vitro* live–dead staining

A549 cells were seeded in 35-mm dishes (3 × 10^5^ cells per dish), grown at 37°C for 24 h, and then co-incubated with 2 ml of different media for 24 h, followed by rinsing three times with PBS (26). For PTT, some A549 cells were incubated with HA-Gd or HA-Gd-DOX for 1.5 h and then irradiated with 1.0 W/cm^2^ NIR light for 5 min. After additional incubation, the cells were rinsed with PBS and stained with calcein-AM and PI. The stained cells were immediately measured by a confocal laser scanning microscope.

### Animal model

All animal manipulations were performed in accordance with the regulations for animal use and care of Zhengzhou University. Female nude mice (20 g) were inoculated by subcutaneous injection of 200 μl of 5 × 10^6^ cells in PBS.

### *In vitro/in vivo* MRI

For the imaging test, HA-Gd aqueous solution (500 μl) containing different gadolinium ion concentrations (0.03, 0.06, 0.09, 0.12, and 0.18 mmol/L) was placed in the EP tube and fixed in the imager, and spin-echo sequence imaging was performed. The T_1_-weighted relaxation rate (r_1_) was obtained by linear fitting of concentration of gadolinium ion and 1/T_1_. Tumor-bearing mice were injected with HA-Gd (10 mg/kg) *via* the tail vein. After 3, 6, 12, and 24 h, T_1_-weighted images were acquired using a 4.7-T clinical MRI scanner (Bruker Icon, Germany).

### *In vivo* photothermal imaging

A549 tumor-bearing mice were randomly divided into two groups. The mice in the experimental group were injected with HA-Gd-DOX (2.19 mg/ml, 100 µl) intravenously, and 24 h later, the tumor area was irradiated with an 808-nm laser with a power density of 1.0 W/cm^2^ for 5 min. The mice in the control group were treated with saline (PBS) administration. Thermal images were recorded by the PTT monitoring MG33 system when tumors were exposed to an 808-nm laser light at a power density of 1.0 W/cm^2^ for 5 min.

### Antitumor effect *in vivo*

When tumor volumes reached about 100 mm^3^, the tumor-bearing mice were divided into seven groups (*n* = 5, each group). Two groups of mice were, respectively, intravenously injected with 100 μl of HA-Gd and HA-Gd-DOX (2.19 mg/ml, [HA-Gd]: 10 mg/kg, [DOX]: 1 mg/kg). After 1 day, the tumor regions of mice were irradiated with NIR light (808 nm, 1.0 W/cm^2^, 5 min). The other five groups of mice were injected with 100 μl of PBS, HA-Gd, DOX, Lipo-DOX, and HA-Gd-DOX ([HA-Gd]: 10 mg/kg, [DOX]: 1 mg/kg), respectively. Similar irradiation treatment was also carried out in the group of NIR. Tumor sizes and body weight were recorded every 2 days during the therapy period. Tumor volumes were calculated *via* the following formula: volume = (tumor length) × (tumor width)^2^/2. After treatment was completed, mice were sacrificed and dissected, and tumors and major organs were collected. The heart organs of each group of mice were weighed and compared to the weight of the mouse. Then, tumors and organs were embedded with paraformaldehyde and stained with hematoxylin and eosin (H&E) for histological analysis.

### Histology analysis

The mice were euthanized. Then, tumor and major organs (heart, lung, liver, spleen, kidney, and tumor) were collected after 14 days, fixed in 4% neutral buffered formalin, embedded in paraffin, cut into 4-μm sections, and stained using hematoxylin and eosin (H&E). Finally, the images of these histological tissue sections were obtained by a confocal laser scanning microscope.

### Biodistribution *in vivo*

The biodistribution of Gd element *in vivo* of tumor-bearing mice was assessed by intravenous injection of HA-Gd-DOX (15 mg/kg). At different time points (0, 1, 3, 5, and 7 days), the mice were euthanized. The heart, liver, spleen, lung, kidney, and tumor were extracted and mixed with aqua regia (V_nitric acid_/V_hydrochloric acid_ = 1:3) for 24 h to obtain a clear solution and then characterized by inductively coupled plasma-mass spectrometry (ICP-MS) to analyze the Gd content.

### Statistical analysis

The significance of experimental results was analyzed by one-way variance (ANOVA) test and two-tailed Student’s *t*-test using the Prism 8.0 software. Probabilities *p* < 0.05 (*), *p* < 0.01 (**), and *p* < 0.001 (***) were marked in each figure, and 0.05 was chosen as the significance level.

## Results and discussion

### Characterization of samples

**Figure 1:**
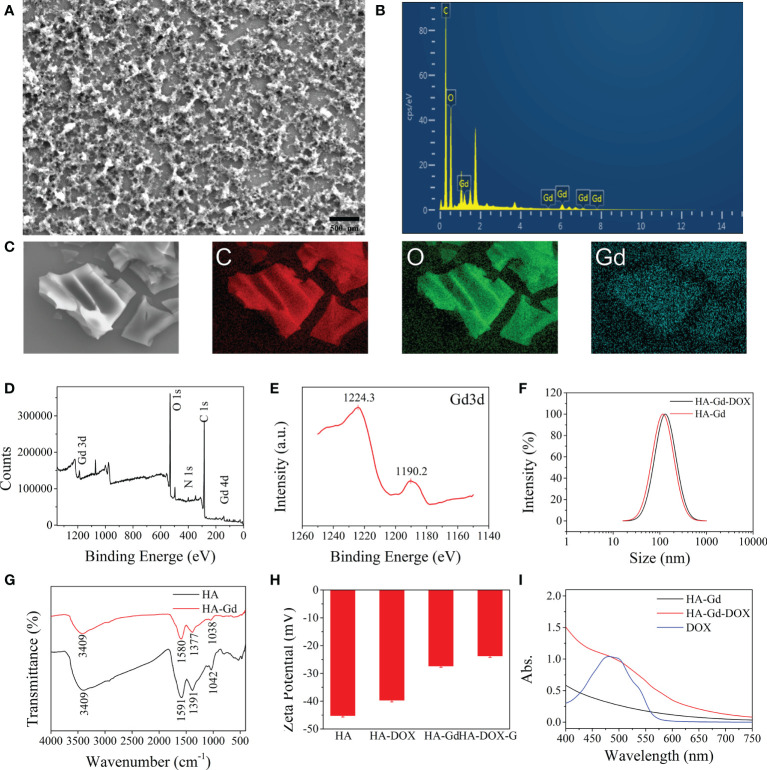
Characterization of the HA, HA-Gd, and HA-Gd-DOX. **(A)** SEM image of HA-Gd. **(B)** EDX of HA-Gd. **(C)** C, O, and Gd mapping images of HA-Gd nanocarriers. **(D)** XPS spectra of HA-Gd. **(E)** XPS spectra of Gd 3d orbit. **(F)** The DLS of HA-Gd and HA-Gd-DOX nanomaterials. **(G)** The FTIR spectra of HA and HA-Gd. **(H)** Zeta potential of HA, HA-DOX, HA-Gd, and HA-Gd-DOX. **(I)** UV-Vis spectra of HA-Gd, DOX, and HA-Gd-DOX.

As shown in **Figure 1A**, HA conjugated with gadolinium ions form the fluffy nano-structure HA-Gd. EDX of HA-Gd (**Figure 1B**) and its mapping results (**Figure 1C**) showed that HA-Gd mainly contains C, O, and Gd elements, and the gadolinium was evenly distributed in the HA-Gd when it was freeze-dried. Additionally, XPS analysis results in **Figure 1D** showed that HA-Gd contained C, O, N, and Gd. The high-energy peak (Gd 3d) and low-energy peak (Gd 4d) are observed at 1,200 eV and around 150 eV, respectively. Meanwhile, the existence of Gd^3+^ ions can be manifested by the 3d peaks at approximately 1,190.2 eV (^3^d_5_) and 1,224.3 eV (^3^d_3_) (**Figure 1E**) (27). Meanwhile, the high-resolution spectrum of Gd 4d (at 140–160 eV) confirmed the successful Gd^3+^ chelation with HA (28). In order to investigate the size distributions of HA-Gd, dynamic light scattering (DLS) was conducted, and the results are shown in **Figure 1F**. The size of the HA-Gd was approximately 116 nm, which ensured the EPR effect for nanoscale biomaterials (60–400 nm) and the accumulation of HA-Gd at the tumor region.

In order to further explore the bonding mechanism between HA and Gd^3+^, FTIR and zeta potential analysis on the composite materials were carried out (**Figures 1G, H**). It can be seen that the extended -OH stretching vibration absorption peak at 2,000–3,750 cm^−1^ on bare HA obviously shrunk and the absorption peak at 1,000–1,600 cm^−1^ on bare HA moved to a low wave number region, which could be attributed to -OH conjugated with Gd ions to form salt. The zeta potential value of HA increased from −45 to −26.5 mV after conjugating with Gd^3+^, further demonstrating that HA and Gd^3+^ formed a stable complex and the negatively charged surfaces could allow them to escape from the clearance by the reticuloendothelial system *in vivo* and could be expected to maintain a long circulation time.

HA-Gd loaded and encapsulated DOX to construct a drug delivery system for antitumor therapy. The UV-Vis absorption spectrum of the HA-Gd-DOX showed a characteristic absorption peak at 480 nm of loaded DOX (**Figure 1I**). The zeta potential value of HA-Gd-DOX was more positive than HA-Gd (**Figure 1F**), indicating that HA-Gd can efficiently encapsulate DOX, and the DLS size distribution of HA-Gd-DOX was 131 nm (**Figure 1H**), which makes HA-Gd-DOX a promising nanodrug delivery system for tumor treatment.

### DOX loading and *in vitro* release

**Figure 2:**
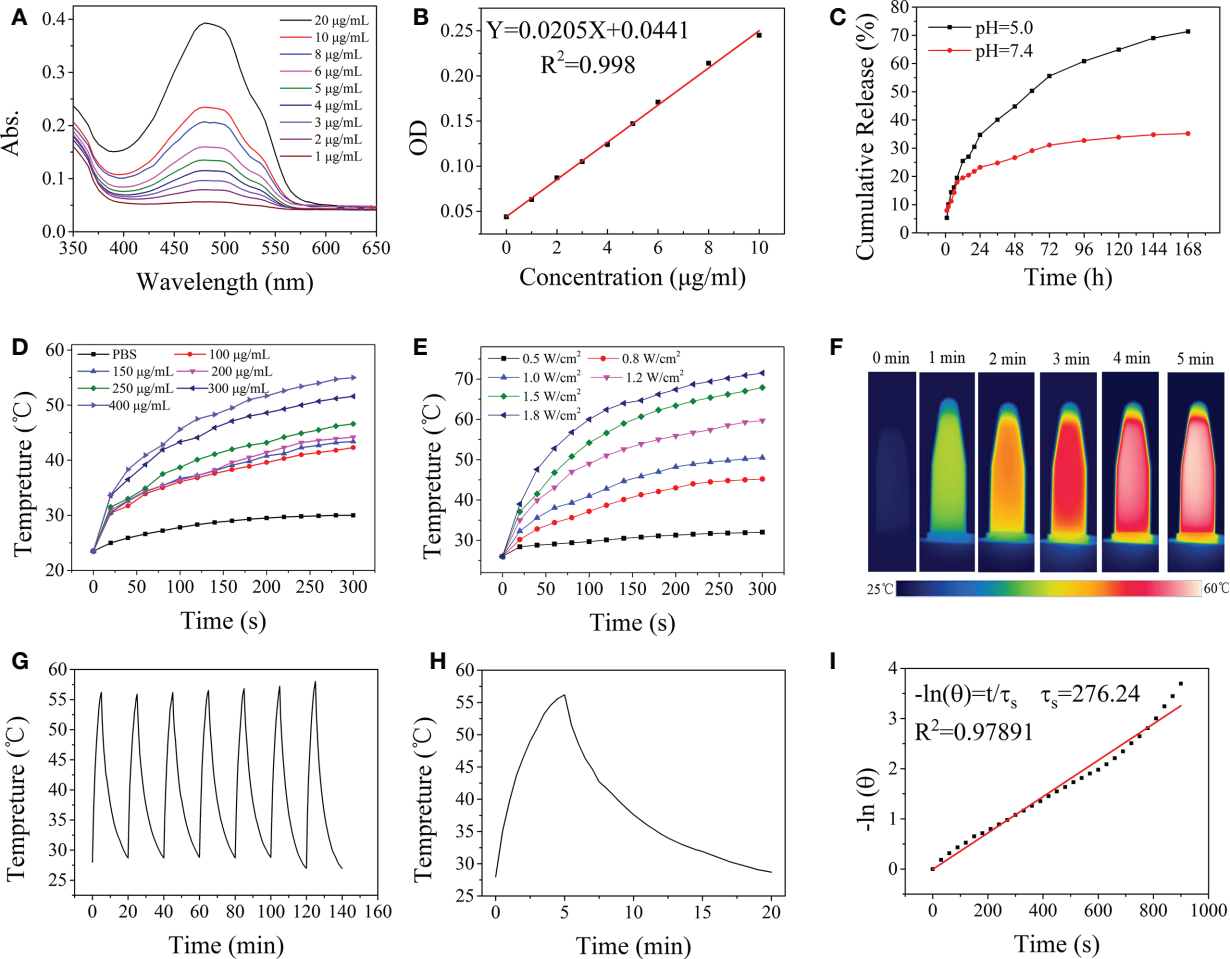
Drug release and photothermal properties of nanodrugs. **(A)** UV-vis absorption spectra of DOX at different concentrations. **(B)** Fitting curve equation of DOX. **(C)** *In vitro* DOX release profile at different pH values of HA-Gd-DOX. **(D)** The temperature elevation of the HA-Gd with different concentrations. **(E)** The temperature elevation of the HA-Gd on different powers of laser. **(F)** Thermal imaging of the HA-Gd with concentrations of 400 μg/ml under an 808-nm laser (1.0 W/cm^2^). **(G)** Temperature change of HA-Gd solution at a concentration of 400 μg/ml over seven laser on/off cycles under 808-nm laser irradiation with a power density of 1.0 W/cm^2^. **(H)** The photothermal conversion results of 400 μg/ml HA-Gd under an 808-nm laser (1.0 W/cm^2^). **(I)** Fitted curve of photothermal conversion efficiency.

The amount of DOX loaded into the HA-Gd nanocarriers was estimated by measuring the absorbance at 480 nm and was calculated using the standard equation (**Figures 2A, B**). About 93% of the added DOX was encapsulated within HA-Gd, and an equilibrium value (93 mg/g) was eventually reached when the drug and HA-Gd were stirred together for 24 h. The high drug encapsulating capacity was attributed to the porous and fluffy structure formed by the conjugation of HA and Gd^3+^ and the negative charges of the HA-Gd.

The drug release profiles of HA-Gd-DOX were assessed under the physiological (PBS, pH 7.4) and acidic conditions (PBS, pH 5.0) to simulate the endo-lysosomal environment at 37°C. As shown in **Figure 2C**, there was a much faster release of DOX at pH 5.0 solutions, thus achieving a high release content of 73%. The continuous release of HA-Gd-DOX for more than 7 days and the high drug encapsulating capacity could be used as a potential DOX cargo to increase the antitumor efficacy of DOX and minimize the damage to normal organs.

### *In vitro* photothermal effect

The photothermal effect of the HA-Gd was explored in detail. The temperature changes of aqueous solutions containing different concentrations of HA-Gd (0, 100, 150, 200, 250, 300, and 400 μg/ml) exposed to an 808-nm NIR laser (1.0 W/cm^2^, 5 min) are shown in **Figure 2D**. Upon 808-nm NIR laser irradiation, the temperature increases of the sample solutions with concentrations of 100, 150, 200, 250, 300, and 400 μg/ml were 18.8, 19.9, 20.7, 22.1, 27.1, and 31.5°C, respectively, and there was a concentration–effect relationship between concentration and temperature. The results showed that the HA-Gd nanocarriers could well convert the laser energy into thermal energy, indicating their potential application for tumor PTT.

The temperature change of aqueous solutions containing 400 μg/ml of HA-Gd exposed to different powers of the 808-nm NIR laser (0.5, 0.8, 1.0, 1.2, 1.5, and 1.8 W/cm^2^) is shown in **Figure 2E**. The temperature of the medium increased rapidly with the increase in laser intensity. The 400 μg/ml HA-Gd solution was irradiated with 1.0 W/cm^2^ and the temperature was raised above 50°C, which was sufficient for PTT (**Figure 2F**). The photothermal stability test of HA-Gd was conducted and the result is shown in **Figure 2G**. The photothermal conversion ability of the HA-Gd did not decrease even after seven cycles. The photothermal conversion efficiency (*η*) was determined using the calculation formula based on previous literature (29) (see the calculation formula in **Supporting Information**). When HA-Gd suspension (400 μg/ml) was exposed in NIR laser (808 nm, 1.0 W/cm^2^) for 5 min and then the light source was turned off, the temperature variation curve is shown in **Figure 2H**. *τ* was determined by the linear relationship between cooling time and -ln (*θ*) (**Figure 2I**) and the photothermal conversion efficiency of HA-Gd was calculated to be 38%. The high photothermal conversion efficiency of HA-Gd could be used for PTT of tumors.

### Cellular uptake

**Figure 3:**
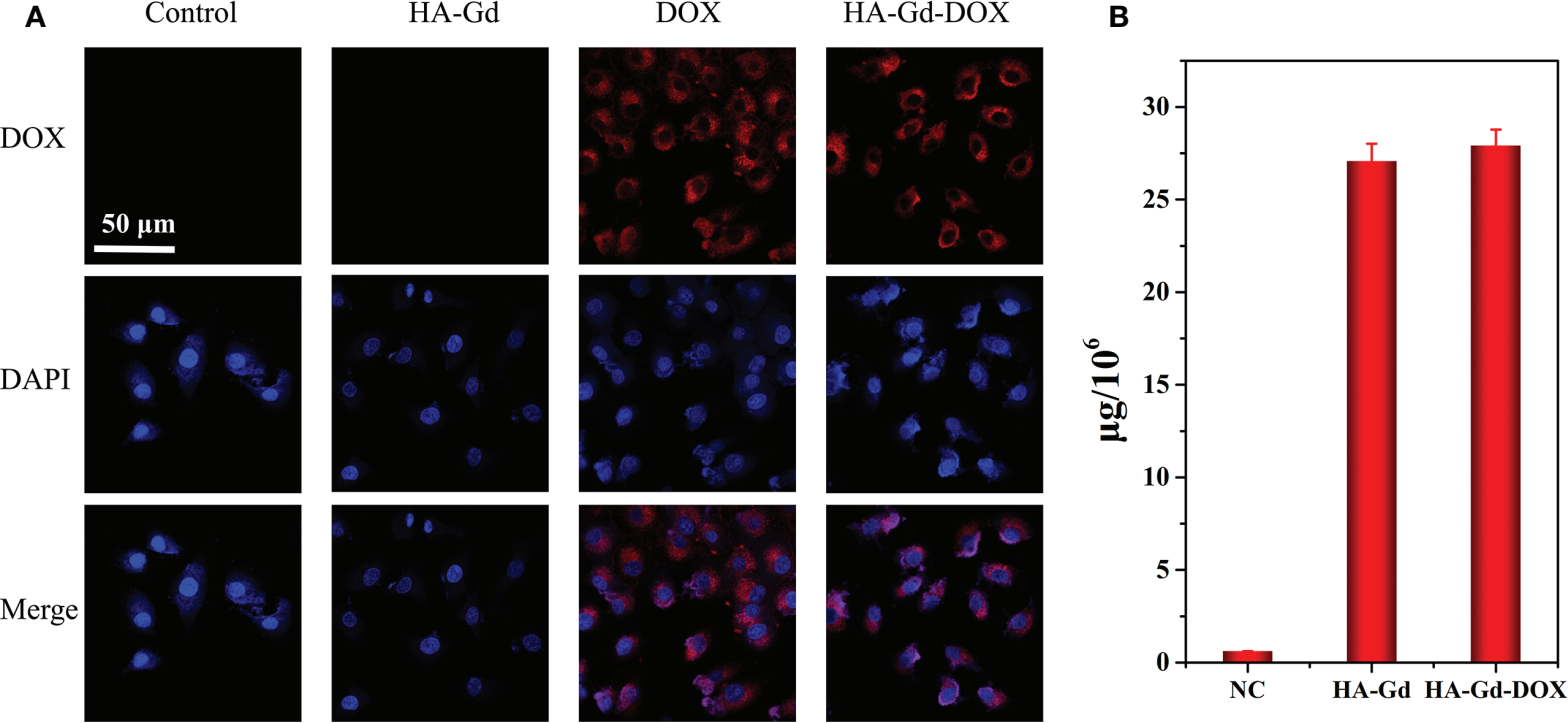
**(A)** CLSM of A549 cells after incubation with HA-Gd, free DOX, and HA-Gd-DOX for 3 h Merge: DOX-DAPI. Scale bars: 50 μm. **(B)** The amount of Gd element in the A549 cell after incubation with PBS, HA-Gd, and HA-Gd-DOX for 24 h.

The cellular uptake behaviors of HA-Gd-DOX in A549 cells were investigated by confocal laser scanning microscopy. As shown in **Figure 3A**, red fluorescence was observed at the cytoplasm within the cells treated with DOX and HA-Gd-DOX for 3 h, while only blue fluorescence in DAPI mode can be observed when treated with HA-Gd, indicating that free DOX and HA-Gd-DOX had been taken up by the A549 cells. To further confirm the HA-Gd-DOX internalization in cells, Gd content was tested by ICP-MS, as shown in **Figure 3B**; 27.1 and 27.9 μg of Gd element per 10^6^ cells after 24 h of HA-Gd and HA-Gd-DOX co-incubation with A549 cells were found, respectively. All the above results indicated that HA-Gd and HA-Gd-DOX could be transported into cells to inhibit cell growth by photothermal therapy/chemotherapy.

### *In vitro* antitumor activity of the HA-Gd-DOX

**Figure 4:**
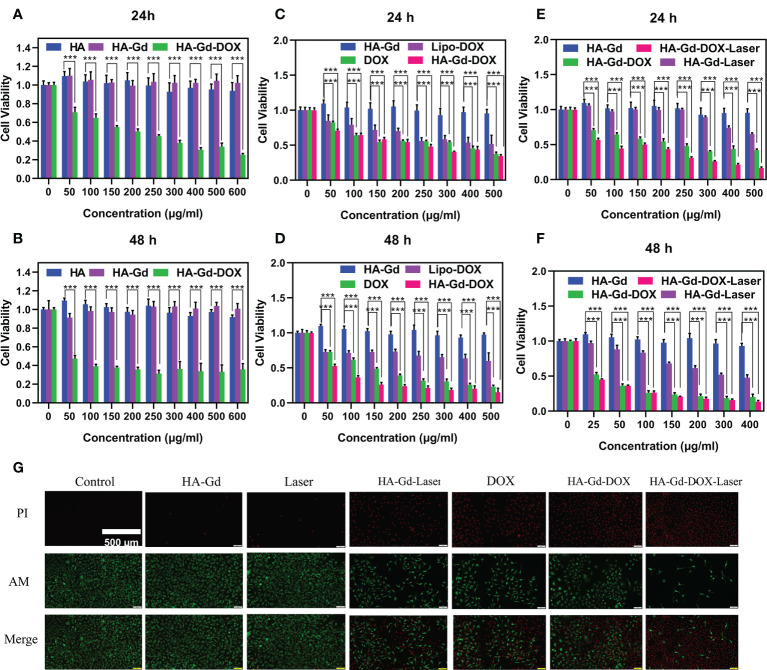
Cell viabilities of A549 treated with different concentrations of HA, HA-Gd, and HA-Gd-DOX for 24 h **(A)** and 48 h **(B)**. Cell viabilities of A549 treated with different concentrations of DOX, Lipo-DOX, and HA-Gd-DOX (corresponding amount of DOX) for 24 h **(C)** and 48 h **(D)**. Cell viabilities of A549 treated with HA-Gd, HA-Gd+laser, HA-Gd-DOX, and HA-Gd-DOX+laser at different concentrations for 24 h **(E)** and 48 h **(F)**. Data are mean ± SD (*n* = 5, *p < 0.05, **p < 0.01, ***p < 0.001; the significance of experimental results was analyzed by two-tailed Student’s *t*-test). **(G)** Live–dead staining images of A549 cells treated with different treatments, the concentration of HA-Gd-DOX is 400 μg/ml, and the laser is irradiated with an 808-nm laser 5 min on 1.0 W/cm^2^. Scale bars are 500 μm.

The biocompatibility of the nanomaterials was an important issue in the fabrication of a drug delivery system. The biocompatibility of the pristine HA and HA-Gd was evaluated using A549 cells by the CCK-8 assay to explore their application prospects in medicine. The results showed that the cell viability of A549 incubated with HA and HA-Gd did not significantly decrease over 24 and 48 h when the concentration increased from 0 to 600 μg/ml compared with the control group, as shown in **Figures 4A, B**. It could be concluded that HA and HA-Gd had good cell cytocompatibility, while the cell viability of A549 gradually decreased with the increased concentration of HA-Gd-DOX. Even at a low concentration of 50 μg/ml, cell activity declined significantly (*p*<0.001) to 70% at 24 h, while the viability of cells showed a significant decline (*p*<0.001) to 45% with the extension of co-incubation time, which was attributed to the gradual internalization of HA-Gd-DOX and the continuous release of DOX in tumor cells. The viability of A549 cells was related to the concentration of HA-Gd-DOX; as the concentration increased, the cell viability gradually decreased to 30% at the concentration of 400 μg/ml.

In order to explore the clinical application of HA-Gd-DOX, we compared the killing effect of HA-Gd-DOX with DOX and Lipo-DOX (contains an equal amount of DOX) on tumor cells. The results are shown in **Figures 4C, D**. At 24 h, with the increasing concentration, the cell viability of A549 incubated with DOX, Lipo-DOX, and HA-Gd-DOX all decreased from 100% to below 50%. However, after 48 h, the cell viability of A549 incubated with HA-Gd-DOX was significantly lower (*p*<0.001) than that of the DOX and Lipo-DOX groups. The above results indicated that HA-Gd-DOX exhibited better antitumor efficacy compared with clinical Lipo-DOX, which also suggested that the HA-Gd-DOX could have an excellent prospective clinical application.

As a novel photothermal agent, the CCK-8 assay was used to evaluate the photothermal treatment effect on A549 cells under photothermal conditions (808 nm laser, 1.0 W/cm^2^, 5 min). As shown in **Figure 4E**, its photothermal therapeutic effect was apparent when the concentrations are above 300 μg/ml. The CCK-8 assay was also used to investigate the chemo-photothermal therapy of HA-Gd-DOX *in vitro*. The cell viability gradually decreased with the increasing concentration of HA-Gd-DOX, while the decrease in cell activity was more obvious under laser irradiation, confirming that the combination of chemotherapy and PTT could more efficiently inhibit cancer cells. In addition, we also studied the effect of chemo-photothermal therapy under the condition of 48 h, and the results are shown in **Figure 4F**. With the extension of co-incubation time, A549 cells treated with HA-Gd-DOX plus laser exposure exhibited more impressive cell inhibition, indicating that the chemo-photothermal combination therapy was superior to monotherapy. HA-Gd-DOX could reduce the dosage of DOX and then further reduce the damage to human body in tumor therapy.

Calcein-AM (green) and PI (red) co-staining experiments were carried out to further verify the antitumor effect of HA-Gd-DOX *in vitro*. Results are shown in **Figure 4G**; the green fluorescence of the control groups, HA-Gd, and laser-alone groups indicated that HA-Gd and laser alone were not toxic to tumor cells. The intense red fluorescence of A549 cells incubated with HA-Gd under near-infrared laser irradiation indicated that HA-Gd could effectively kill tumor cells under laser irradiation. When incubated with DOX and HA-Gd-DOX, a bigger red fluorescence field demonstrated that the death rate of cells increased because DOX entered the cell’s nucleus to further destroy the DNA structure. More importantly, when cancer cells were incubated with HA-Gd-DOX under NIR laser irradiation, more intense red fluorescence meant a significant increase in cell death rate. These results came from the fact that under NIR laser irradiation, HA-Gd-DOX constantly released DOX and gathered hyperthermia, enabling chemo-photothermal combination to efficiently kill tumor cells.

### MRI *in vivo*

**Figure 5:**
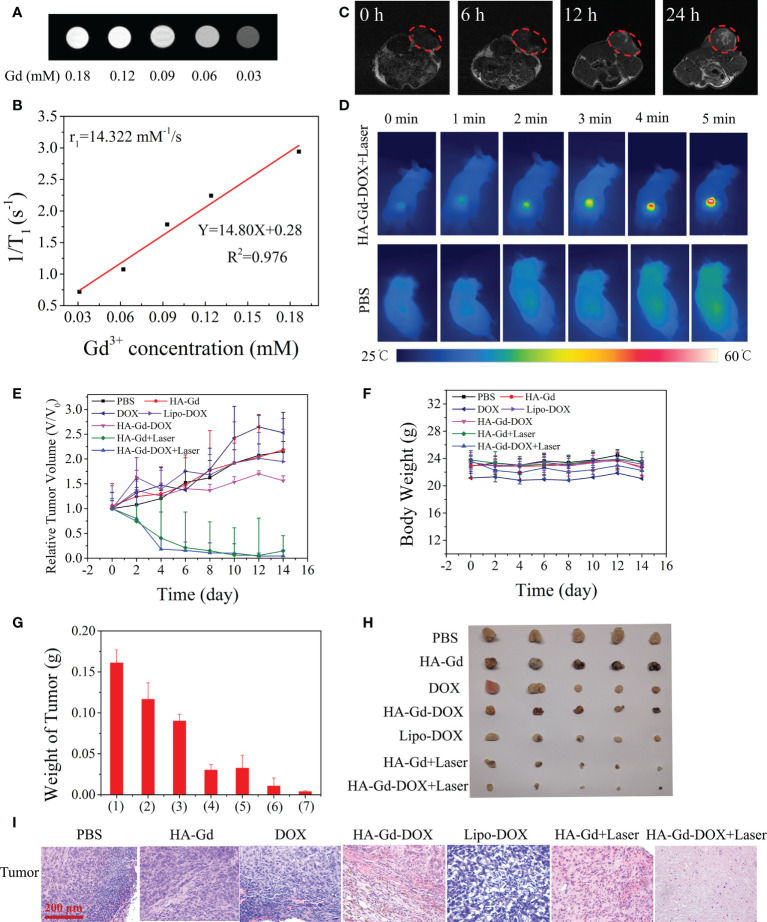
**(A)** T_1_-weighted MR images. **(B)** T_1_ relaxation rates (r_1_) of HA-Gd-DOX at different concentrations. **(C)** *In vivo* T_1_-weighted MR images of a tumor-bearing mouse after injection 0, 6, 12, and 24 h of HA-Gd-DOX (15 mg/kg). **(D)** Infrared thermal images of tumor-bearing mice treated with PBS and HA-Gd-DOX under irradiation with an 808-nm laser at different time periods. **(E)** Tumor volume variation curves and **(F)** body weight variation curves of tumor-bearing mice treated with different treatments. Data are expressed as mean ± SD (*n* = 5). **(G)** The weights of excised tumors after 14 days of various treatments: (1) PBS; (2) HA-Gd; (3) DOX; (4) HA-Gd-DOX; (5) Lipo-DOX; (6) HA-Gd+Laser; and (7) HA-Gd-DOX+Laser. **(H)** Digital photographs of the excised tumors 14 days after treatment from five mice in each group. **(I)** H&E staining images of the tumors collected 14 days after various treatments.

The MRI properties of HA-Gd-DOX dispersions with different Gd concentrations were tested. **Figure 5A** shows the progressively brighter signal intensity in relation to Gd concentration, suggesting that it could be used as a contrast agent for MRI. The transverse relaxivity (r_1_) of the HA-Gd-DOX was measured to be 14.322 mM^−1^ s^−1^ (**Figure 5B**). The aggregation behavior in tumor of HA-Gd-DOX and the MRI efficiency were investigated in tumor-bearing mice models. T_1_-weighted MR images of tumor-bearing mice after injection of HA-Gd-DOX at different time intervals (0, 6, 12, and 24 h) are shown in **Figure 5C**. The T_1_-weighted MR signal at 12 h after injection in the tumor site was obviously detected, and the T_1_-weighted MR signal gradually improved after 24 h, indicating that HA-Gd-DOX gradually accumulated in the tumor region.

### *In vivo* photothermal effect

The photothermal effect of HA-Gd-DOX *in vivo* was investigated by an infrared thermal camera. A549 tumor-bearing mice were intravenously injected with HA-Gd-DOX solutions (200 μl, 1.5 mg/ml) and exposed to an 808-nm laser illumination for 5 min at 24 h post injection, with PBS as the control group. As shown in **Figure 5D**, the temperature of tumor rapidly increased to 55.8°C after intravenous injection of HA-Gd-DOX at a power density of 1.0 W/cm^2^ for 5 min. However, the tumor temperature of mice treated with PBS increased by only 0.9°C. These results suggested that HA-Gd-DOX could be used as an excellent photothermal agent for efficient PTT of solid tumors *in vivo*.

### Anticancer effect *in vivo*

*In vivo* combination therapy experiments were performed in A549 tumor-bearing mice using HA-Gd-DOX as PTT and chemotherapeutics. The mice were randomly divided into seven groups: (1) PBS as the control group, (2) DOX, (3) HA-Gd, (4) HA-Gd-DOX, (5) lipo-DOX, (6) HA-Gd **+** 808-nm laser irradiation, and (7) HA-Gd-DOX **+** 808-nm laser irradiation. The changes in tumor volume were recorded every 2 days. The results are shown in **Figure 5E**. The tumor volume of the PBS, HA-Gd, and free DOX groups increased rapidly, indicating that HA-Gd and DOX alone had no significant tumor suppression effect. Lipo-DOX exhibited therapeutic effect in the initial stage, whereas with the extension of time, its therapeutic effect gradually decreased. Compared with the tumor volume of the free DOX and Lipo-DOX group, the tumor volume treated with HA-Gd-DOX was relatively smaller. The HA-Gd **+** 808-nm group had obvious effective against tumor, convincing its PTT for tumor. The optimal tumor suppression of the HA-Gd-DOX + 808-nm group indicated its powerful chemo-photothermal therapeutic effect. In this study, no weight changes were detected in the treatment group, indicating no significant systemic toxicity (**Figure 5F**). Tumor weight at the end of therapy (**Figure 5G**) and digital photos of tumors excised from representative mice (**Figure 5H**) also showed the best therapeutic effect of HA-Gd-DOX + 808-nm laser irradiation. To further clarify the mechanism of therapy, histological analysis, mainly hematoxylin and eosin (H&E) staining, was performed on the tumor sections after different treatments (**Figure 5I**). H&E staining results showed that the tumors treated with HA-Gd-DOX and laser irradiation received the most massive cell remission and high cell apoptosis, which could be attributed to a chemo/PTT combination effect.

### *In vivo* biodistribution and toxicity evaluation

**Figure 6:**
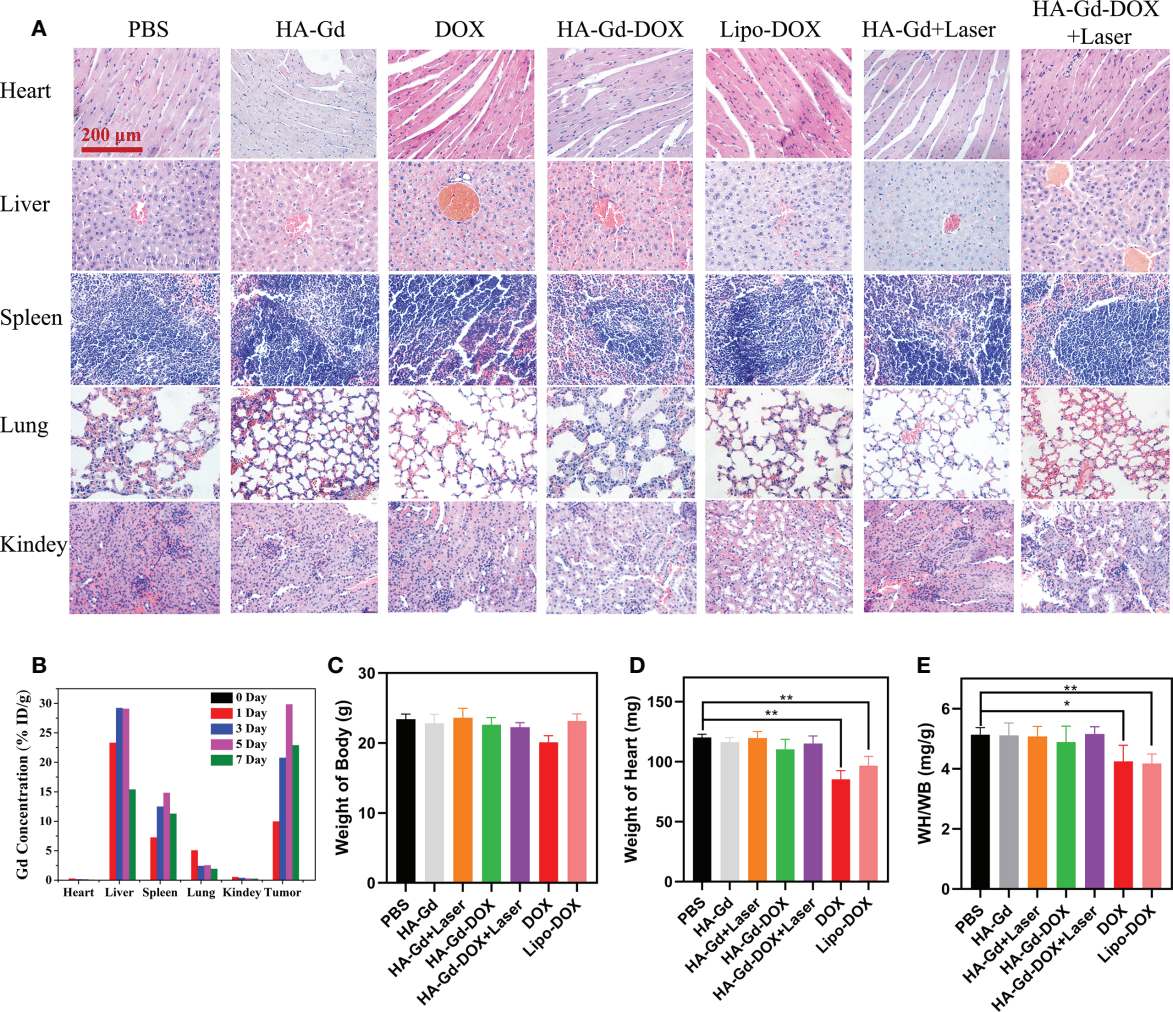
**(A)** Histopathologic examination of the tissues from tumor-bearing mice after different treatments. **(B)** Distribution of Gd element in tissues after tail vein injection of HA-Gd-DOX (15 mg/kg). Body weight **(C)**, heart weight **(D)**, and cardioplasmic ratio **(E)** of tumor-bearing mice with different treatments after 14 days (*n* = 5, *p < 0.05, **p < 0.01; the significance of experimental results was analyzed by two-tailed Student’s *t*-test).

In the treatment of tumors, the safety of NPs was an important aspect to examine. Histological analysis was carried out to examine the safety for major organs of mice treated with HA-Gd, DOX, Lipo-DOX, HA-Gd + laser, HA-Gd-DOX, and HA-Gd-DOX + laser, and the PBS control group; the results are shown in **Figure 6A**. No noticeable signal of tissue damage was observed in the heart, liver, lung, spleen, and kidney, confirming HA-Gd-DOX’s low systematic toxicity during cancer therapy. To further understand the biodistribution and metabolic pathways of HA-Gd-DOX in mice, Gd content in the major organs of mice treated with HA-Gd-DOX was measured by ICP-MS. As shown in **Figure 6B**, after 24 h of injection, Gd was mainly distributed in the spleen and liver. Gd gradually accumulated in the spleen and liver over time. Cumulative gadolinium content reached its maximum on the fifth day and thereafter gradually decreased, suggesting that HA-Gd-DOX metabolized primarily through the spleen and liver in mice. The minimal concentrations of Gd in the lungs and kidneys during the week of treatment suggested the safe use of HA-Gd-DOX. Most curious to us, gadolinium was almost undetectable in the heart, which made us investigate the effect of HA-Gd-DOX in reducing DOX’s cardiotoxicity.

Cardiac weight coefficient was a commonly used indicator for cardiac toxicology tests in mice. The weight and heart mass of mice in different treatment groups were recorded to calculate the cardiac weight coefficients, and the results are shown in **Figures 6C–E**. The body weight and heart mass of mice in the DOX group had both reduced, and the heart mass of mice in the Lipo-DOX group had also reduced compared to the control group. The calculated results showed that the heart weight coefficient of both DOX and Lipo-DOX groups was reduced, while the heart weight coefficient of HA-GD-DOX and HA-GD-DOX + laser groups was unchanged compared with the control group, which indicated that HA could protect the heart from the cardiac toxicity of DOX.

## Conclusion

In summary, a novel nanocarrier based on the conjugation of HA with gadolinium ions was facilely fabricated to form an HA-Gd nanocarrier. HA-Gd exhibited excellent biocompatibility and dispersity, high photothermal conversion efficiency, excellent photothermal stability, and high DOX loading capacity with pH-responsive release properties. To exploit the intrinsic physical nature of HA-Gd, MRI was performed on tumor-bearing mice, demonstrating efficient accumulation of nanomaterials at tumor sites. Compared with Lipo-DOX, HA-Gd loading DOX inhibited tumor growth more effectively through the combination of photothermal therapy and chemotherapy, and less toxicity and side effects were observed in treated animals. The results showed the great potential of HA as a novel vector for developing more drug carriers with desirable functions for clinical anticancer therapy. Additionally, its uptake path in cells, transport process, antitumor mechanism on the cell and animal, and the metabolic process *in vivo* should be further investigated to explore its clinical application.

## Data availability statement

The original contributions presented in the study are included in the article/**Supplementary Material**. Further inquiries can be directed to the corresponding authors.

## Ethics statement

The animal study was reviewed and approved by Ethics Committee of Zhengzhou University.

## Author contributions

JK and YL contributed equally to this research paper. JK and WM initiated the project. JK, YL, YD, LL, TQ, SL, MW, and WD performed the experiment. JK and YL wrote and finalized the manuscript. All authors reviewed and commented on the entire manuscript. All authors contributed to the article and approved the submitted version.

## Funding

This work was supported by the National Natural Science Foundation of China (grant nos. 82073168 and 31570917), the Fundamental Research Funds for the Universities of Henan Province (grant no. NSFRF220439), the Henan Science and Technology Research Project (grant no.222102310399) and Key scientific research projects of colleges and universities in Henan Province(23B350001).

## Conflict of interest

The authors declare that the research was conducted in the absence of any commercial or financial relationships that could be construed as a potential conflict of interest.

## Publisher’s note

All claims expressed in this article are solely those of the authors and do not necessarily represent those of their affiliated organizations, or those of the publisher, the editors and the reviewers. Any product that may be evaluated in this article, or claim that may be made by its manufacturer, is not guaranteed or endorsed by the publisher.
