# Supplementary material for: A novel vector for magnetic resonance imaging-guided chemo-photothermal therapy for cancer
Source: Front Oncol. 2022 Oct 17;12:972082. doi: 10.3389/fonc.2022.972082 (PMC9618661; doi:10.3389/fonc.2022.972082)
Supplement: Supplementary file 1 [file DataSheet_1.docx]

**Supporting Information**

**A Novel Vector for Magnetic Resonance Imaging Guided** **Chemo-Photothermal Therapy for Cancer**

**Ji chuan Kong^1^**†***, Yang Li^1^** †**, Wang Ma^1,2^*,Yu rong Du^2^, Lei Liu^2^, Tian tian Qu^2^, Shuo shuo Liu^1^ , Meng li Wang^1^, Wei Dou^1^**

^1^ Medical College, Henan Polytechnic University, Jiaozuo, China,

^2^Department of Oncology, The First Affiliated Hospital of Zhengzhou University, Zhengzhou, China

*** Correspondence:**

**Ji chuan Kong**

E-mail: [kongjichuan@hpu.edu.cn](mailto:kongjichuan@hpu.edu.cn)

**Wang Ma**

E-mail:[doctormw@zzu.edu.cn](mailto:doctormw@zzu.edu.cn)

The photothermal conversion efficiency (η) was calculated **via** the following formula :

$$\eta=\frac{hS{\Delta T}_{max}-Q_{S}}{I(1-{10}^{-A})}=\frac{hS{(\Delta T}_{max}-{\Delta T}_{maxs})}{I(1-{10}^{-A})} (1)$$

$$hS=\frac{m_{s}C_{S}}{\tau} (2)$$

Where △T_max_ represents the temperature change of sample at the maximum steady-state temperature; △T_maxs_ represents the temperature change of solvent at the maximum steady-state temperature; I is NIR laser power density; A represents the 808 nm absorbance of sample; C_s_ and m_s_ are the heat capacity and mass of solvent, respectively;$\tau$ is the sample system time constant, which can be determined by the linear curve fitting of temperature cooling time *vs* its ln $\frac{\Delta T}{{\Delta T}_{max}}$ (θ =$\frac{\Delta T}{{\Delta T}_{max}}$ ).


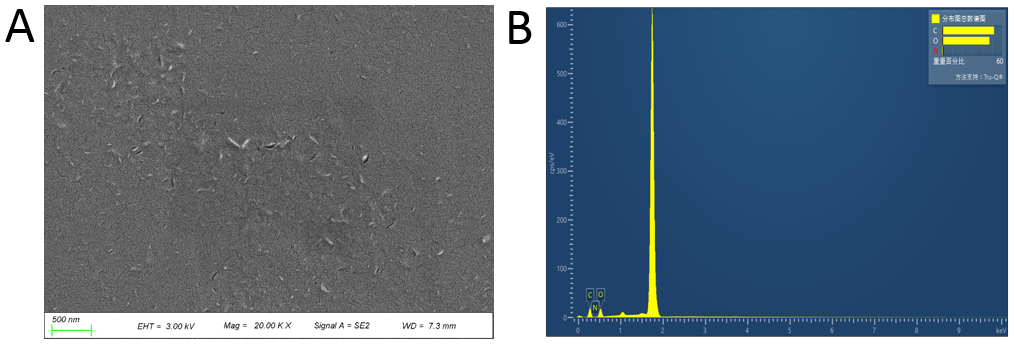


**FIGURE S1** | **(A)** SEM image of HA.**(B)** EDX of HA





**FIGURE S1** | XPS spectra of Gd 4d orbit
